# Supplementary material for: Chromothripsis during telomere crisis is independent of NHEJ, and consistent with a replicative origin
Source: Genome Res. 2019 May;29(5):737–49. doi: 10.1101/gr.240705.118 (PMC6499312; doi:10.1101/gr.240705.118)
Supplement: Supplemental Material [file supp_gr.240705.118_Supplemental_file_1.zip › contigs/annotated_contigs/DB37/contig.2.DB37_length_356_mean_cov_3.20786516854.docx]

**DB37_length_356_mean_cov_3.20786516854**

```
TTTGTATATATACCCAGAGATGGGATTGCTGGATCATATGGCAATTCTATGTTCAAATTTTGAAGAACCTCCATACTGTATTTTAGATG
 >chr18:67084800-67085050 + E=3e-139
GAAATAATTTTTATTTCCATCAACAGTGTGCAAACATTCTCTTTCCTTTGCATCCTCGCCAGCATTTATTATTTTCTGTCTTCTTAATA

GTAGCCATTCTAATTGGGGTGAGATAATATCTCATTGTGGTTTCTATTTGCATTTCCCAAATGATTAG|TGAT|CATTATACATTGCAT
                                                                     >chr18:67084147-67
TATTGTATCAAAATATTACATGTATTCCATAAATATGGACAACTATTATGTATCCATAAACATAAAAAAATTTAAAAATTAAAAATAGC
084257 - E=9e-55
AT
```
